# An International Comparison of Female and Male Students' Attitudes to the Use of Animals

**DOI:** 10.3390/ani1010007

**Published:** 2010-12-09

**Authors:** Clive Phillips, Serdar Izmirli, Javid Aldavood, Marta Alonso, Bi Choe, Alison Hanlon, Anastasija Handziska, Gudrun Illmann, Linda Keeling, Mark Kennedy, Gwi Lee, Vonne Lund, Cecilie Mejdell, Veselinas Pelagic, Therese Rehn

**Affiliations:** 1 Centre for Animal Welfare and Ethics, University of Queensland, Australia; 2 Department of History of Veterinary Medicine and Deontology, University of Selcuk, Turkey; E-Mail: sizmirli@selcuk.edu.tr; 3 Faculty of Veterinary Medicine, University of Tehran, Iran; E-Mail: sja@ut.ac.ir; 4 Department of Animal Production, University of Leon, Spain; E-Mail: marta.alonso@unileon.es; 5 Department of Bioethics, The Catholic University of Korea, Seoul, Korea; E-Mail: bi.choe@samsung.com; 6 School of Agriculture, Food Science and Veterinary Medicine, University College Dublin, Ireland; E-Mail: alison.hanlon@ucd.ie; 7 AgroWeb Farm Animal Welfare Network Co-ordinator for Macedonia, Skopje, Macedonia; E-Mail: anastasijahandziska@yahoo.com; 8 Institute of Animal Science, Department of Ethology, Prague, Czech Republic; E-Mail: illmannova.gudrun@vuzv.cz; 9 Department of Animal Environment and Health, Swedish University of Agricultural Sciences, Skara, Sweden; E-Mails: linda.keeling@hmh.slu.se (L.K.); Therese.Rehn@hmh.slu.se (T.R.); 10 Department of Life Sciences, Anglia Ruskin University, Cambridge, UK; E-Mail: Mark.Kennedy@anglia.ac.uk; 11 Graduate School of Veterinary Medicine, Konkuk University, Seoul, Korea; E-Mail: labvet@konkuk.ac.kr; 12 National Veterinary Institute, Oslo, Norway; E-Mail: cecilie.mejdell@vetinst.no; 13 Fond SFS Center for Education, Research and Consulting in Agriculture, Novi Sad, Serbia; E-Mail: pelagicv@eunet.rs

**Keywords:** animals, attitudes, Asia, Europe, gender, welfare

## Abstract

**Simple Summary:**

We surveyed university students in 11 Eurasian countries for their attitudes to animals, using an internet-based questionnaire to which 1,902 female and 1,530 male student responded from 102 universities. Across countries female students had greater concern for animal welfare and rights than males, but especially so in more gender empowered countries. One contributing factor appeared to be the greater association of females than males with pets, and a possible outcome was greater female avoidance of meat consumption, especially red meat.

**Abstract:**

Previous research has demonstrated that in households where the male partner is more dominant, there is convergence in male and female attitudes towards animals, whereas if the female partner is empowered they exhibit greater empathy towards animals than the male partner. We tested this theory of ‘female empowered empathy’ internationally in a survey of female and male students' attitudes towards use of animals, conducted in 11 Eurasian countries: China, Czech Republic, Great Britain, Iran, Ireland, South Korea, Macedonia, Norway, Serbia, Spain and Sweden. Gender empowerment was estimated for each country using the Gender Empowerment Measure designed by the United Nations. The survey was administered via the internet in universities within countries, and 1,902 female and 1,530 male student responses from 102 universities were received. Respondents rated the acceptability of 43 major concerns about human use of animals, and the importance of 13 world social issues, including animal protection, environmental protection and sustainable development. Females had greater concern for animal welfare and rights than males. There was a positive correlation between the Gender Empowerment Measure and the ratio of female to male concern for animal welfare and rights, but not for other world issues. Thus in countries where females were more empowered, principally Sweden, Norway and Great Britain, females had much greater concern than males for animal issues, whereas in other countries the responses of males and females were more similar. Across countries female students were more likely to avoid meat and less likely to avoid eggs, milk and seafood than male students, and were more likely to have kept pets than males. Females rated cats as more sentient than males did. The results demonstrate that females have greater concern for animal welfare and rights than males, and that this is more likely to be expressed in countries where females are relatively empowered, suggesting that ‘emancipated female empathy’ operates across countries as well as at a local level.

## 1. Introduction

It is generally perceived that women are more sensitive than men to animals welfare, and there is ample evidence of females being more empathetic toward animal issues [1,2,3,4,5,6,7]. Phillips and McCulloch [5] investigated the beliefs of students of different nationalities on both animal sentience and attitudes towards the uses of animals, and found that female students had more concern than males for animal welfare and rights. Gender did not influence the students' beliefs about the degree of sentience of a range of animal species. In another survey, the use of animals in research was more opposed by women than men across a range of countries, including Great Britain, USA, Japan, France and Germany [8]. Further evidence of females' greater interest in animal issues is provided by the composition of animal rights activist organisations, who according to a study by Einwohner [9] are predominantly female, at least in the USA.

The difference between cohabiting females and males in their attitude to animals may depend on the extent of social dominance in partnerships. In a survey of college students in the southern USA, Hyers [10] found a positive relationship between Social Dominance Orientation (a measure of support for a hierarchical social structure and inequality favouring their group over others) and endorsement of the use of animals for “luxury” and “non-luxury” purposes. This relationship may also exist in different countries, influenced by the socio-political situation and cultural heritage, including religion. For example, some countries have a long history of religious instruction supporting both male dominance in society and the use of animals, whereas others do not. A survey by Baxter and Kane [11] conducted in five countries (USA, Australia, Canada, Norway and Sweden) found that women with high social, economic and interpersonal dependence on men had attitudes more similar to men. Generally societies with high levels of gender equality are also more supportive of environmental protection, suggesting that these two societal characteristics, attitudes towards animals and towards environmental protection, at least coexist and may be interdependent [12].

As part of an international survey of attitudes to animals, we examined whether social dominance differences between countries influence attitudes towards the use of animals, by surveying the student population in a range of Eurasian countries. Previous research has identified a range of indices that can be used to measure attitudes to different aspects of use of animals from this survey [13], and we utilised these to obtain a better understanding of male and female attitudes to animal welfare and rights in different nations. This may promote understanding of their different needs in relation to teaching about animals, or to teaching with the aid of animals, and also to animal product consumption.

## 2. Methods

### 2.1. Survey Method

The survey method was approved by the Human Ethics Committee of the University of Queensland and has been described in full previously [13]. In brief, a call was distributed through relevant organizations, e.g. International Society for Applied Ethology, for volunteer academic collaborators to organize the survey in their country. Suitable collaborators volunteered in 21 countries worldwide, but those in 9 countries dropped out over the course of the project, leaving 12 countries as a convenience sample. Subsequently Portugal was also excluded because of the low response rates to the survey. Those remaining represented a broad spectrum of cultures and geographical regions of Europe and Asia (China, Czech Republic, Great Britain, Iran, Ireland, South Korea, Macedonia, Norway, Serbia, Spain and Sweden). In all cases except Norway and Sweden, where access to entire student populations by e-mail was possible, collaborators organized a team of student volunteers in a sample of universities in their country. Universities were selected at random if possible, but in some countries a convenience sample was used. Student volunteers approached students at a central location in the university (not related to any subject area) and asked them if they would take part in a social survey. This was anticipated to avoid the potential bias of students interested in animals being more likely to complete a survey on animals if asked to do so. A pilot survey was conducted at the University of Queensland to test the methodology for recruitment of questionnaire respondents, out of 100 students that were approached, 50% indicated a willingness to take part in the survey and 17 completed questionnaires were returned. In total responses were received from students at 103 universities in the main survey, with the target number of respondents in each country being related to the population. If they agreed to take part in the survey, the students were asked to give their e-mail address to the volunteer.

The survey format and content was discussed and agreed by all collaborators, and the survey was then translated by the collaborators into the native language, who were most familiar with the animal welfare terminology used. Some of these translated versions were translated back and changes made in the case of discrepancies, and in all cases the survey meaning and translation was checked by a third party for accuracy and consistency of meaning, in conjunction with the collaborator. Weblinks to the survey were then distributed to the students by e-mail with an accompanying password. In the case of Norway and Sweden the initial approach to students was by e-mail.

Students were asked questions relating to demographics, food consumption preferences and about the acceptability of 43 animal issues and importance of thirteen world issues. In this paper we report only the influence of gender on animal and world issues, the effects of country on food consumption and on animal/world issues being reported elsewhere (respectively, [14,15]). The 43 animal issues were originally based on the major concerns about our use of animals. These are (1) the use of animals; (2) animal integrity; (3) killing animals; (4) animal welfare; (5) experimentation on animals; (6) changes in animal genotypes; (7) animals and the environment; (8) societal attitudes towards animals. Each concern was represented by approximately five questions. The questions were chosen by the project team, including country collaborators, to be of international, not regional concern, and to be mutually exclusive. They were as follows [13]:

Use of Animals
  AI 1 Keeping animals for the production of food or clothing
  AI 2 Keeping animals as pets
  AI 3 Keeping animals for the education of the public in zoos, wildlife parks *etc.*
  AI 4 Using animals for work
  AI 5 Using animals for entertainment or sports
Animal Integrity
  AI 6 Operations on animals to improve their health
  AI 7 Decoration of animals, such as dying or cutting their hair for aesthetic reasons
  AI 8 Desexing by hormone implants
  AI 9 Removal of a body part, such as tail docking, or declawing
  AI 10 Marking animals by branding or ear notching
  AI 11 Removal of dead tissue, such as hair/wool removal or foot trimming
Killing Animals
  AI 12 Killing young animals that are dependent on their parents
  AI 13 Allowing animals to experience pain during slaughter
  AI 14 Using animals for products after their natural death
  AI 15 Killing animals when they are seriously injured or ill
  AI 16 Euthanizing healthy and unwanted pets because of overpopulation
Animal Welfare
  AI 17 Depriving animals of their needs for food and water
  AI 18 Depriving animals of an appropriate environment to rest, including shelter
  AI 19 Inflicting pain, injury or disease on animals
  AI 20 Not providing sufficient space, proper facilities and company needed for animals
  AI 21 Subjecting animals to conditions and treatment which cause mental suffering
Experimentation on Animals
  AI 22 Observing animal behaviour in an experiment
  AI 23 Experiments to improve animal welfare or health
  AI 24 Medical experiments using animals to improve human health
  AI 25 Testing cosmetics or household products on animals
  AI 26 Operating on living animals for the benefits of human medicine research
Changes in Animals' Genotypes
  AI 27 Increasing animals' reproductive or productive capabilities by genetic changes, e.g., cows producing more milk
  AI 28 Increasing animals' health or disease resistance by genetic changes
  AI 29 Creating farm animals that feel happy with little stimulation and have little desire to be active
  AI 30 Genetic selection of pet animals, such as dogs and cats, to increase their rarity, potential for showing or pedigree value
  AI 31 Genetic modification of crops grown for animal foods
Animals and the Environment
  AI 32 Killing animals because they are not native to the area in which they live
  AI 33 Killing wild animals to stop the spread of diseases that could affect humans
  AI 34 Controlling wildlife populations by killing
  AI 35 Controlling animal populations by sterilization
  AI 36 Destroying the habitat of endangered animal species
  AI 37 Destroying the habitat of non-endangered animal species to develop and promote urbanization or crops to feed humans
Societal Attitudes Towards Animals
  AI 38 Sacrifice of animals in religious rites
  AI 39 Considering some animal species as sacred or good luck symbols or totems
  AI 40 Considering some animal species as evil or bad luck
  AI 41 Parents displaying cruel treatment of animals in front of their children
  AI 42 Inflicting pain or injury on animals as part of cultural traditions
  AI 43 Cloning animals for human benefit

Students were asked to rate the acceptability of the practices described on a Likert scale of 1, extremely unacceptable, to 5, extremely acceptable. Thirteen questions were asked concerning major world social issues, and students were asked to give their opinion about how important each was, on a scale of 1, not important, to 7, extremely important [13]. The questions were:

WI 1 Animal protection
WI 2 Professional ethics
WI 3 Capital punishment
WI 4 Environmental protection
WI 5 Racial equality
WI 6 Genetic engineering
WI 7 Equality for lesbian, gay, bisexual and transgender
WI 8 Human cloning
WI 9 Human euthanasia
WI 10 Reducing poverty
WI 11 Sustainable development
WI 12 Women's rights
WI 13 Peace and security

Students were also asked to rank a list of animals in relation to their capacity for feeling (hereafter termed sentience): cat, cattle, chicken, chimpanzee, dog, dolphin, fish, horse, human infant, octopus, pig and rat.

### 2.2. Statistical Analysis

Responses to the 43 Animal Issues have been previously subjected to a factor analysis [13] to reduce the number of output variables. Sample sizes of all nations were weighted to be roughly equal, which reflects the assumption that the opinions of each nation count equally. The method of extraction of factors was by principal components analysis with Varimax rotation. Factors with loadings of less than 0.2 were excluded. The components that were significantly correlated for each factor were examined, and a summary title was provided for each factor, which was changed from those originally proposed by Meng [13] to increase clarity. This analysis identified factors that represented views on Animal Welfare, Animal Rights, Unnatural Practices on Animals, Killing Animals, Animals in Experimentation, Wildlife and Animals as Spiritual Symbols. The questions on which the indices were based, and formula for creating the index scores from the 1–5 rating by each student, were as follows (listing the questions in declining order of importance):

Animal Welfare Index = 98.8 − 6.2 A18 − 5.2 A13 − 4.3 A17 − 2.7 A12 + 2.5 A2 − 1.6 A9 − 0.5 A5
Animal Rights Index = 104 − 2.6 A8 − 2.4 A1 − 1.9 A12 − 1.8 A3 − 1.6 A13 − 1.6 A10 − 1.6 A5 − 1.5 A4 − 1.2 A9 − 1.1 A7 − 0.8 A.2
Unnatural Practices on Animals Index = 116 − 4.0 A28 − 3.9 A30 − 3.4 A27 − 3.0 A31 − 2.5 A3 − 2.2 A7 − 2.3 A36 − 1.9 A8 + 1.9 A12 + 1.9 A10 − 1.7 A2
Killing Animals Index = 107 − 3.6 A14 − 3.4 A22 − 3.1 A11 − 3.1 A4 − 2.8 A15 + 2.6 A36 − 2.3 A32 − 2.2 A8 − 2.0 A1 − 2.0 A12 + 2.0 A20
Animals in Experimentation Index = 115 − 5.2 A24 − 4.0 A26 − 3.5 A23 + 3.3 A36 + 2.2 A8 + 2.2 A30 + 1.9 A37 − 1.9 A43 − 1.8 A33 − 1.8 A1 + 1.7 A18
Wildlife Index = 92 − 4.9 A37 − 4.4 A33 − 4.1 A36 − 3.2 A34 + 2.7 A22 − 2.6 A16 + 2.2 A14 − 2.0 A20 + 1.9 A25 − 1.8 A2 − 1.8 A9
Animals as Spiritual Symbols Index = 108 − 6.5 A39 − 5.6 A40 − 4.9 A2 − 3.1 A6 − 2.3 A42 − 2.2 A9 − 1.8 A23 + 1.8 A29 − 1.8 A38 + 1.5 A35 − 1.3 A28

A similar factor analysis was conducted for the World Issues that summarised attitudes to these issues in one value, containing the following questions (again in order of declining importance):

World Issues Index = 0.17 W4 + 0.16 W10 + 0.16 W11+ 0.16 W12 + 0.16 W5 + 0.15 W13 + 0.15 W1 + 0.15 W2 + 0.1 W7 + 0.09 W3 + 0.09 W6 + 0.08 W9 + 0.04 W8

To examine the influence of demographic variables, including gender, on the eight indices, binary logistic regression, ANOVA, and Chi square analyses were compared in terms of their effectiveness for modelling the data. Because binary logistic regression and ANOVA gave similar and more discriminating results than Chi square, and the data either approximated a normal distribution or could be manipulated to a normal distribution, ANOVA was selected because of its flexibility for modelling the data. Following an initial analysis the residual data distribution was examined and where necessary transformed. This was only required for one variable, the Animal Welfare index, and a squared function gave the necessary approximately normal distribution. The model for data responses included nation, ethnic group (nested within nation), gender, level of education, area of study, place of residence, religious affiliation, food avoidance, reasons why food was avoided and animal protection organisation participation. Only gender is considered in this paper.

Relationships between measures of female empowerment and the female to male ratio of responses in the indices were examined. The Gender Empowerment Measure (GEM) and Gender-Related Development Index (GDI) [16] were compared for their relationship to the ratio of female to male responses for these indices. The GEM was chosen because it focuses on agency, as indicated by political participation and decision-making power, economic participation/power, and command over economic resources. Specifically the GEM is a composite measure which includes (i) the proportion of seats held by women in national parliaments; (ii) the percentage of women in economic decision-making positions, including administrative and managerial positions, as well as professional and technical occupations; and (iii) the female share of income [16]. In contrast, the GDI relates more to human development and less to gender inequality. Linear regression of the three different GEM components: (1) Politic participation (proportion of women in the Lower or Single House in parliament), (2) Senior workplace roles (Income, ratio of female to male GDP per capita), and (3) Economic participation (proportion of women in senior positions: legislators, senior officials, managers, professional workers and technical workers) on the relevant indices was performed for each country. These variables were all normally distributed by the Anderson-Darling test. No Gender Empowerment Measure values were available for China and Serbia, or for Serbia for Income, therefore data for these countries were omitted for the specific regressions. The expenditures (ratio of male to female) measured in our study were compared to Income as possible measures of gender effects on economic activity, but found to be too variable. All analyses were conducted using the statistical packages Minitab 15 and SPSS 15.

## 3. Results

A total of 3,432 responses (1,902 female and 1,530 males) were obtained from an estimated 16,700 students that provided their e-mail addresses in 103 universities (Table 1).

### 3.1. Animal Indices

Of all the indices tested for the relationship to gender, only the Animal Welfare and Rights indices were significantly affected by gender overall (Table 2), with more concern for both of these issues by female students. However, there was a gender × country interaction for all of the animal indices, except Killing Animals and Animals as Spiritual Symbols, indicating that there were significant differences between genders in some countries (Table 3). In Norway, Sweden and Great Britain there existed the greatest differences between female and male responses, with on average females having a mean index score of 64 and males 57 for those indices significantly affected. In other countries a higher index score for females over males was restricted to only a few indices and the differences were relatively small. There were no countries or indices where males had significantly greater scores than females.

**Figure 1:**
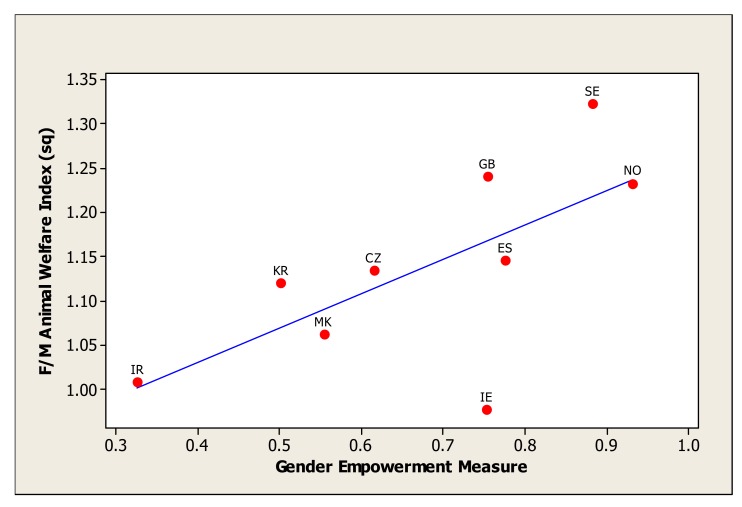
Ratio of female to male (F/M) scores on the Animal Welfare Index^2^ to the Gender Empowerment Measure (GEM). CZ = Czech Republic, GB = Great Britain, IR = Iran, IE = Ireland, KR = South Korea, MK = Macedonia, NO = Norway, ES = Spain, SE = Sweden.

**Figure 2:**
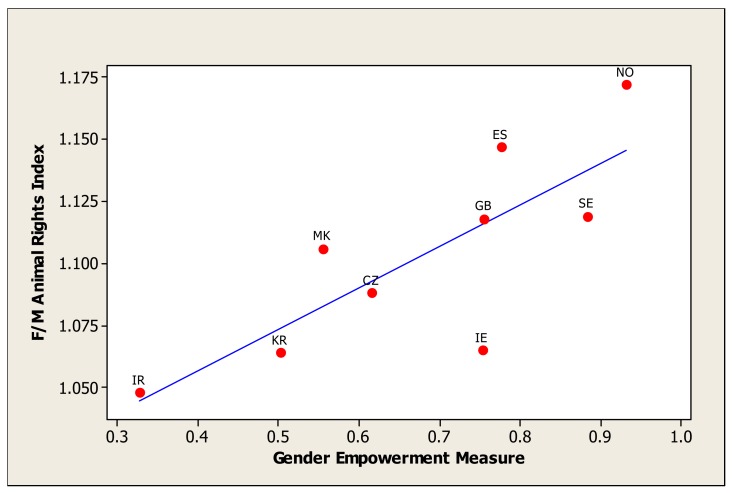
Ratio of female to male (F/M) scores on the Animal Rights Index to the Gender Empowerment Measure (GEM). CZ = Czech Republic, GB = Great Britain, IR = Iran, IE = Ireland, KR = South Korea, MK = Macedonia, NO = Norway, ES = Spain, SE = Sweden.

Individual country's scores for the GEM component are presented in Table 4. There were significant relationships between GEM and the ratio of female to male scores on the Animal Welfare, Animal Rights and Animal Experimentation indices, with the strongest relationship being with the Animal Rights Index (Table 5). Overall, Political Participation and Senior Workplace Roles were the important components in the GEM in explaining this relationship, not Economic Participation. For the Animal Rights index ratios this was primarily due to the Political Participation component of the GEM; for the Animal Welfare index ratios it was due to both Political Participation and Senior Workplace Roles. The relationship between female to male (F/M) Animal Welfare Index^2^ and the Gender Empowerment Index is shown in Figure 1, and it is evident that there is a high correlation, but that Ireland is an outlier. The same trend is evident for the relationship between F/M Animal Rights Index and the Gender Empowerment Index (Figure 2), with a good correlation except for Ireland.

### 3.2. Sentience

The overall order of sentience (mean rank and SED mean) was similar for males and females (Table 6). Mean values (±SE) for females and males together were as follows: human infant 10.7 ± 0.043 > chimpanzee 9.7 ± 0.040 > dog 9.5 ± 0.030 > Dolphin 8.6 ± 0.044 > Cat 7.7 ± 0.035 > Horse 7.2 ± 0.034 > Cattle 5.5 ± 0.034 > Pig 5.2 ± 0.039 > Rat 4.8 ± 0.045 > Chicken 3.8 ± 0.032 > Octopus 2.7 ± 0.038 > Fish 2.6 ± 0.039. However, female students rated pigs and dolphins as less sentient than males did, and human infants and cats were rated as more sentient by females than males.

### 3.3. Consumption of Animal Products

There were differences between males and females in the avoidance of animal products (Table 7). Females were more likely to avoid meat than males, and the proportion of female vegetarians was three times that of males. Females were much more likely to cite their health as the main reason for avoiding eating or using animal products, whereas males were more likely to cite the environment and, to a lesser extent, animal suffering. A small proportion of students cited religious instruction as the reason, and most were males. Female students were more likely than male students to avoid meats, particularly the red meats, beef, lamb and to some extent pork (Table 8). They were less likely to avoid eggs, milk and seafood than male students.

### 3.4. Pet Keeping and Animal Protection Society Membership

Female students (n = 1894) had approximately 50% more years of pet keeping (0.45) than males (n = 1524) (0.30), (SED 0.012, *P* < 0.001). They were also more likely to be key members of animal protection societies (2.9%) than male students (1.8%), and to very often (7.4% compared with 4.8% for males) or sometimes (39% compared with 32% for males) support animal protection societies (*P* < 0.001).

### 3.5. World Issues

Females rated the rights of women as a much more important issue than males did (Table 9). They also rated equality for lesbian, gay, bisexual and transgender (LGBT), human euthanasia, animal protection and professional ethics more highly than males, and peace and security, racial equality and environmental protection received marginally higher ratings. There was no significant difference between males and females in their rating of the importance of capital punishment, genetic engineering, human cloning, reducing poverty and sustainable development.

Expressed as a rank order, World Issues were similar for male and female students, except that females elevated the rights of women from 8th to 4th place. For both males and females animal protection was ranked the second most important World Issue, behind environmental protection. The overall order of World Issues for females (with mean rank and SED mean) was environmental protection (6.6 + 0.03) > animal protection (6.4 + 0.04) > sustainable development (6.1 + 0.03) > rights of women (5.9 + 0.04) > reducing poverty (5.9 + 0.04) peace and security (5.9 + 0.03) > racial equality (5.9 + 0.04) > professional ethics (5.7 + 0.04) > equality for LGBT (5.0 + 0.06) > capital punishment (4.9 + 0.06) > genetic engineering (4.5 + 0.05) > human euthanasia (4.4 + 0.06) > human cloning (3.7 + 0.07). The overall order of World Issues for males (and mean rank and SED mean) was environmental protection (6.5 + 0.03) > animal protection (6.2 + 0.04) > sustainable development (6.0 + 0.03) > reducing poverty (5.9 + 0.04) > peace and security (5.8 + 0.03) > racial equality (5.7 + 0.04) > professional ethics (5.5 + 0.04) > rights of women (5.1 + 0.04) > capital punishment (4.8 + 0.06) > equality for LGBT (4.7 + 0.06) > genetic engineering (4. 6 + 0.05) > human euthanasia (4. 1 + 0.06) > human cloning (3.7 + 0.07).

## 4. Discussion

This survey used novel techniques for recruitment of students across a major part of Europe and Asia, which is a logistical problem that has made this type of research difficult in the past. We acknowledge that the method of contacting students may have influenced the level of participation or engagement—direct email or being approached on campus. In addition for students approached on campus transcription errors or students giving erroneous email addresses was another potential problem that may have reduced the response rate. However, with recent development of opportunities for electronic transfer of survey forms and responses, such techniques offer the possibility to investigate global attitudes much more easily than in the past. Because of the scale and breadth of the survey, co-ordination of collaborators' activities assumed a major importance. One area in which difficulties could potentially be experienced was translation as it was impossible with so many countries to arrange back-translation, as is usually advocated for such cross-cultural research [17,18]. However, we attempted to ensure consistency of meaning by effective checking of the translated version and discussion of discrepancies with the translator. Another potential limitation of the study was that it utilised student respondents, who may vary between countries in their representativeness of the general population.

The greater concern of females than males for animal welfare has been enunciated previously by several authors [1,2,4,5,6]. In a similar but smaller survey to the current one, female students had more concern for both animal suffering during life and the reverence for animal life than males [5]. Heleski *et al.* [4] found greater empathy for agricultural animal welfare in females than males. As well as animal welfare, we found a similar increase in concern for animal rights by females, compared with males. Peek *et al.* [19] suggests that this may be because of the relational role of women in society, having primary responsibility for nurturing, empathy and care towards others. Patriarchal domination was recommended as an alternative influence and suitable subject for future study [20]. Cultural feminist theory suggests that women make moral judgements based on relations rather than universal standards of right and wrong. Kruse [21] suggests that males support a primarily Darwinian view of animals, in which the natural world is exploited and controlled, whereas females have primarily a Romantic view of animals, with greater affection for them and concern for their ethical treatment.

We did not find any overall difference between males and females in attitudes to other animal issues, for example animal experimentation. However, Pifer *et al.* [8] found that women were more opposed to use of animal in research than men in 15 nations. Wells and Hepper [1] found that adult males disagreed less with the use of animals by humans than adult females, and women opposed medical testing of animals more strongly than men. A survey conducted in New Zealand also found that females were more concerned about the use of animals in research, testing and teaching than males [22]. The similarity between males and females in our survey in attitude to experimentation with animals may derive from the greater concern that female students had for their health, compared with males. This was evident in their eating preferences, with more females avoiding meat because of the possible impact on their health.

Female concern for animal welfare is also evidenced by veterinary student surveys. In a study by Paul and Podberscek [2], female students rated themselves as having significantly higher levels of emotional empathy with animals than did the male students. There was also a significant interaction between gender and year of study, with the female students maintaining relatively high levels of empathy throughout the three years, whereas the male students showed lower levels of empathy in their later years [2]. In a study by Serpell [6], female veterinary students displayed greater concern for possible instances of animal suffering than males, and prior experience with different animals, as well as rural background and farm experience, were also associated with attitude differences. Seventy-two percent of students reported that their interactions with animals (especially pets) had strongly influenced the development of their values [6]. Therefore it is possible that in our survey the much greater pet ownership by females than males was a driving force behind their attitude to animal welfare and rights. Greater ownership of pets by females than males in our survey is supported by the finding of Vidovic *et al.* [23] that females had a greater attachment to pets than males.

In the present study, it was found that there was no significant difference in perception of sentience of any animal species as a result of gender, except cats, dolphins, human infants and pigs. Phillips and McCullough [5] found no gender differences in attributed sentience of any animal in their survey, but dolphins and cats were not included. The greater attribution of sentience by females to human infants is expected because of their generally increased contact and nurturing responsibilities than males. Greater attributed sentience for cats in females than males is probably because cats tend to be preferred by females and dogs by males, and cats are seen as having more feminine characteristics, with dogs displaying masculine characteristics of activity, dominance, aggression [24]. More females than males identify themselves with cats, whereas dog appreciation is gender non-specific [24]. This gender difference in cat appreciation may also be evidenced by the tendency of females towards greater grief after cat death [25] and greater willingness of female French veterinarians to provide analgesia to cats [26], although these were both also evident for dogs. Females in our survey credited pigs with less sentience than males did, which may be because of a perceived lack of cleanliness in pigs, common in many cultures, with female respondents being disinclined to credit pigs with high levels of sentience as a result. Because our survey asked students to rank species by sentience, if females attributed greater sentience to human infants and cats, other species had to fall in rank. According to a survey of Prokop and Tunnicliffe [27], girls were less favourably inclined than boys to animals that may pose a threat, danger or disease risk to them, which may incline them towards cats and away from pigs that are perceived to pose a disease risk.

The Eurobarometer is one of the only instruments used to explore gender differences in animal welfare status and buying habits. According to the 2005 Eurobarometer [28], males rated the welfare and protection status of pigs and dairy cows more positively than female, whereas they were similar for laying hens. The 2007 Eurobarometer [29] found that more females (63%) displayed a “willingness to change usual place of shopping in order to buy welfare friendly produce” than males (59%). In our survey animal welfare was clearly a consideration for both male and female students in food choices. The 2005 Eurobarometer [28] asked participants “When you purchase meat (*i.e.*, poultry, beef, pork, fish) do you think about the welfare/protection of the animals from which these meat products have been sourced”. More females responded positively to this question (49%) than males (38%). Our survey found that males were less likely to avoid some meats than females, but those that did were more likely to do so because of animal suffering and the environment than females, who were more likely to do so because of their health.

Neumark-Sztainer *et al.* [30] found that most adolescent vegetarians are female (81%), which was similar to the 78% in our survey. Others found that females tend to characterise meat and meat-eating experiences negatively, and red meat-eating is more common among males than females [31,32]. In the present study, female were also more likely than males to avoid meat, particularly the red meats, beef, lamb and to some extent pork. Clearly males have fewer ethical concerns about red meat consumption. This acceptance of the use of animals for human benefit appears to be greater in males, as they also have less concern about accepting xenografts from animals, which are mainly pigs [33]. The avoidance of red meat by females probably relates mainly to their greater concern for their health, with red meat consumption being associated with cardiovascular and cancer risks, in particular. However, some females may be aware that livestock that produce red meat are sometimes kept in inhumane conditions. The fact that eggs were avoided less by females than males, whereas there is much publicity given to inhumane treatment of laying hens, suggests that animal suffering may not be the main driver of food avoidance in women. Women could believe that they are able to eat eggs that are produced to high welfare standards, given the widespread availability of eggs that are sold under this premise. It is also possible that women are more anthropomorphic than men and bestow humanlike qualities more easily on mammals that are phylogenetically more similar to humans, deterring them from eating these animals more than less similar animals, such as hens. In the present study, females were found to be more sensitive to animal protection, professional ethics, environmental protection, racial equality, equality for LGBT, human euthanasia, rights of women and peace and security than males. A survey of Baxter and Kane [11] which was conducted in the USA, Australia, Canada, Norway and Sweden, demonstrated that men hold less egalitarian gender role attitudes than do women. In our study students in Norway, Sweden and Great Britain were most likely to display gender inequality in attitudes to animal welfare, with female students being generally more concerned about animal issues than male students. Women in Sweden, Norway and Great Britain have high Gender Empowerment Scores, relative to other EU27 countries, which includes measures of the proportion of women in parliaments and other senior positions with high incomes [34]. Women in these countries are relatively more economically independent than women in other countries in our survey; they are not so likely to marry and when they do it is at an older age [11]. The dependence of women on men in other countries encourages them to adopt masculine attitudes [11], which may explain why attitudes to animal issues were least similar for students in Sweden, Norway and Great Britain. Our regression models of GEM and F/M ratio of responses on the Animal Welfare and Rights Indices showed that Ireland was an outlier, but the sample was very small, less than any other country. It is highly likely that the gender disparity would be different for different issues, indeed we found this in our study. However, it is unwise to read too much into one point on the graph, *i.e.*, one country.

The Human Development Report Office proposed both a Gender-related Development Index (GDI) and the Gender Empowerment Measure (GEM) as means of monitoring international progress in the development of women's capabilities [35]. In spite of the limitations of GEM; we favoured it because it reflects a higher level of gender empowerment and is positively associated with a more equal division of labour in the household [36]. The GEM has been criticised for measuring inequality mainly among the most educated and economically advantaged and failing to include important non-economic dimensions of decision-making power, both at the household level and over women's own bodies and sexuality [37]. In addition the data used for measuring disparity in income is problematic [16] and in our survey it failed to discriminate adequately between countries. However, despite this we believe that GEM remains the most appropriate measure for the purposes of this international comparison.

## 5. Conclusions

Female students have greater concern for animal welfare and rights than males, especially in countries where there is a low level of dependence of women on men. The increased concern of women contributes towards, but is not the main driver for, greater avoidance of meat, especially red meat. The longer association of females with pets than males may play a significant role in developing their attitude towards animals.

## Figures and Tables

**Table 1:** Response rates from individual countries.

Country	Initial Response Rate	Email Addresses	Completed Questionnaires	% Female Responses	Secondary Response Rate ^1^	Overall Response Rate ^2^
China	77	8211	1018	48.9	12	10
Czech Republic	67	1779	939	57.0	53	35
Great Britain	77	259	54	66.7	21	16
Iran	25 ^3^	573	133	31.6	23	6 ^3^
Ireland	34	600	45	60.0	8	3
South Korea	NA	1984	309	36.9	16	NA
Macedonia	NA	492	101	51.5	21	NA
Norway	NA	382	261	79.3	68	3 ^3^
Serbia	NA	469	207	64.1	44	NA
Spain	75	1741	162	57.1	9	7
Sweden	NA	287	204	82.3	71	5 ^3^

1 Completed questionnaires/Number of email addresses;

2 Completed questionnaires/number of students approached;

3 Approximate values;

NA: Not available.

**Table 2:** Differences between males and females in mean acceptability of issues relating to indices for Animal Welfare, Animal Rights, Unnatural Practices on Animals, Killing Animals, Animals in Experiments, Wildlife, Using Animals as Spiritual Symbols. High values mean greater concern for animals.

	Animal Welfare Squared	Animal Welfare	Animal Rights	Unnatural Animal Practices	Killing Animals	Animals in Experimentation	Wildlife	Animals as Spiritual Symbols
Female	7041	83.6	64.7	67.7	50.9	71.4	59.2	52.2
Male	6531	80.2	61.9	66.5	49.8	70.3	57.5	53.7
SED	37.48	–	0.32	0.57	1.44	0.48	0.44	0.49
*P* value	0.003	–	0.01	0.54	0.47	0.50	0.27	0.40

**Table 3:** Relationship between gender (F = female, M = male) and country in indices for Animal Welfare, Animal Rights, Unnatural Practices on Animals, Killing Animals, Animals in Experiments, Wildlife, Using Animals as Spiritual Symbols. High values mean greater concern for animals.

	**Animal Welfare^2^**	**Animal Rights**	**Unnatural Animal Practices**	**Animals in Experimentation**	**Wildlife**	**Animals as Spiritual Symbols**	**Killing Animals**

China	F 4867	F 57.6	F 50.3	F 56.7	F 68.1	F 43.1	F 57.9
	M 4340	M 55.1	M 51.8	M 53.6	M 65.0	M 44.5	M 55.7

Czech Rep.	F 5973	F 55.9	F 63.5	F 62.5	F 52.2	F 39.5	F 48.7
	M 5267	M 51.4	M 59.4	M 54.7	M 50.7	M 41.1	M 45.4

Gr. Britain	F 6775	F 61.4	F 63.3	F 57.8	F 63.0	F 40.3	F 41.7
	M 5459	M 54.9	M 59.6	M 53.2	M 51.7	M 39.8	M 43.3

Iran	F 6151	F 55.0	F 33.2	F 53.1	F 66.3	F 60.8	F 50.4
	M 6102	M 52.5	M 33.7	M 50.4	M 61.6	M 63.3	M 50.4

Ireland	F 6004	F 60.6	F 72.0	F 62.1	F 58.4	F 44.6	F 48.3
	M 6143	M 56.9	M 66.0	M 54.1	M 56.7	M 37.1	M 43.8

S. Korea	F 5594	F 53.4	F 56.0	F 51.4	F 58.3	F 62.8	F 50.3
	M 4988	M 50.2	M 54.5	M 45.8	M 57.6	M 64.0	M 47.8

Macedonia	F 5780	F 61.2	F 56.3	F 54.1	F 47.7	F 46.6	F 70.3
	M 5441	M 58.2	M 51.9	M 55.2	M 48.0	M 50.2	M 65.7

Norway	F 6230	F 57.1	F 68.6	F 64.1	F 52.9	F 47.3	F 38.8
	M 5052	M 48.7	M 62.3	M 54.4	M 45.2	M 48.4	M 35.4

Serbia	F 5936	F 63.4	F 61.6	F 54.6	F 54.01	F 47.5	F 68.2
	M 5646	M 57.5	M 59.8	M 47.0	M 49.1	M 54.2	M 61.4

Spain	F 6274	F 59.9	F 67.9	F 51.5	F 62.3	F 51.9	F 52.4
	M 5472	M 52.2	M 62.2	M 43.3	M 61.3	M 51.9	M 44.5

Sweden	F 6302	F 52.5	F 64.4	F 66.7	F 48.4	F 50.0	F 31.4
	M 4760	M 46.9	M 66.7	M 56.1	M 39.4	M 50.1	M 31.8

SED, Gender × Country interaction	173.1	1.19	2.01	1.74	1.49	1.77	1.69

P Value, Gender × Country Interaction	0.001	0.01	0.02	0.004	0.004	0.43	0.25

**Table 4:** Scores for the Gender Empowerment Measure and its different components for each country, (1) Politic Participation (proportion of women in the Lower or Single House in parliament), (2) Senior Workplace Roles (Income, ratio of female to male GDP per capita) and (3) Economic Participation (proportion of women in senior positions: legislators, senior officials, managers, professional workers and technical workers).

	Political Participation ^1^	Senior Workplace Roles ^2^	Economic Participation ^3^	Gender Empowerment Measure
China	20.3	0.64	NA	NA
Czech Rep.	17.0	0.51	40.0	0.62
Gr. Britain	19.7	0.65	39.5	0.76
Iran	4.1	0.38	23.0	0.33
Ireland	13.3	0.51	40.0	0.75
S. Korea	13.4	0.46	22.5	0.50
Macedonia	19.3	0.48	40.5	0.55
Norway	37.9	0.75	39.5	0.93
Serbia	12.0	NA	NA	NA
Spain	36.0	0.50	39.5	0.78
Sweden	45.3	0.81	41.0	0.88

1 Proportion of women in the Lower or Single House in parliament;

2 Ratio of female to male GDP per capita;

3 Proportion of women in senior positions: legislators, senior officials, managers, professional workers and technical workers.

**Table 5:** Relationship between the Gender Empowerment Measure, and its components of Political participation, Senior workplace roles and Economic participation, and the ratio of female to male scores for the indices for Animal Welfare, Animal Rights, Unnatural Practices on Animals, Killing Animals, Animals in Experiments, Wildlife, Using Animals as Spiritual Symbols and World Issues for each country.

	Animal Welfare^2^	Animal Rights	Unnatural Animal Practices	Animals in Experimentation	Wildlife	Animals as Spiritual Symbols	Killing Animals	World Isssues

GEM								

*Coefficient*	0.39	0.17	0.62	0.24	0.26	0.45	0.07	−0.09
*R^2^*	44.2	62.3	8.7	46.3	31.2	8.5	4.5	6.0
*P Value*	0.05	0.01	0.44	0.04	0.12	0.45	0.59	0.53

Political participation								

*Coefficient*	0.006	0.002	0.001	0.002	0.003	0.001	0.001	−0.000
*R^2^*	64.7	56.5	0.6	27.7	23.1	0.4	1.5	0.4
*P Value*	0.003	0.008	0.82	0.10	0.13	0.85	0.72	0.85

Senior workplace roles								

*Coefficient*	0.66	0.17	0.44	0.22	0.52	0.42	−012	−0.03
*R^2^*	70.8	27.9	4.3	18.7	62.7	4.1	6.9	0.3
*P Value*	0.002	0.12	0.57	0.21	0.01	0.58	0.47	0.87

Economic participation								

*Coefficient*	0.006	0.003	−0.006	0.002	0.003	−0.005	0.002	−0.002
*R^2^*	13.7	39.6	2.0	6.3	6.7	1.7	5.9	5.7
*P Value*	0.33	0.07	0.72	0.51	0.50	0.74	0.53	0.54

**Table 6:** The scores for perceptions of sentience in specified animals by female and male respondents. High values mean greater concern for animals.

	H. Infant	Chimpanzee	Dog	Dolphin	Cat	Horse	Cattle	Pig	Rat	Chicken	Octopus	Fish
Female	10.3	9.6	9.3	8.6	9.0	7.7	4.7	5.3	4.9	3.8	2.6	2.3
Male	9.9	9.4	9.3	9.0	8.7	7.6	4.8	5.7	4.9	3.9	2.7	2.2
SED	0.09	0.08	0.07	0.09	0.07	0.07	0.07	0.08	0.09	0.07	0.08	0.08
*P* Value	0.02	0.30	0.46	0.04	0.01	0.77	0.22	<0.01	0.99	0.63	0.57	0.68

**Table 7:** Food avoidance and the reasons for it in male and female students.

*Food Avoidance*	Female	Male	*P* Value
No avoidance (%)	41	58	<0.001
Avoid certain meat (%)	53	40	
Vegetarian (%)	6	2	
Vegan (%)	0.4	0.3	
Total number of responses	1902	1530	

*Reasons for Food Avoidance*			

Animal suffering (%)	14	21	<0.001
Environment (%)	34	44	
Religious instruction (%)	3	9	
My health (%)	49	26	
Total number of responses	769	487	

**Table 8:** Differences between male (M) and female (F) respondents in animal products that they used regularly.

	Beef/Veal	Lamb	Pork	Poultry Meat	Eggs	Milk	Seafood	Other Animal Products
	F M	F M	F M	F M	F M	F M	F M	F M
Eating (%) ^†^	41 55	16 23	53 62	69 73	77 70	75 61	41 36	06 03
Non-Eating ^†^ (%)	59 45	79 65	41 26	31 27	18 18	20 28	54 53	89 85
Total responses	1869	2020	2020	1869	2020	2020	2020	2020
*P* value	<0.001	<0.001	<0.001	0.05	<0.001	<0.001	<0.001	<0.001

† Non-respondents (not listed) comprise the difference between eating + non-eating and 100%

**Table 9:** The effects of gender on mean scores for World Issues.

	Female	Male	SED	*P* Value
Animal Protection	6.39	6.16	0.04	0.001
Professional Ethics	5.70	5.49	0.04	0.002
Capital Punishment	4.92	4.85	0.06	0.53
Environmental Protection	6.63	6.53	0.03	0.05
Racial Equality	5.87	5.67	0.04	0.005
Genetic Engineering	4.45	4.56	0.05	0.31
Equality for LGBT	5.03	4.70	0.06	0.006
Human Cloning	3.75	3.67	0.07	0.61
Human Euthanasia	4.39	4.12	0.06	0.02
Reducing Poverty	5.91	5.90	0.04	0.97
Sustainable Development	6.12	6.01	0.03	0.09
Rights of Women	5.94	5.12	0.04	<0.0001
Peace and Security	5.90	5.77	0.03	0.009
